# Ivan Illich and convivial palliative care

**DOI:** 10.1017/S1478951524001937

**Published:** 2025-01-21

**Authors:** Aldis H. Petriceks

**Affiliations:** Beth Israel Deaconess Medical Center, Boston, MA, USA; Harvard Medical School, Boston, MA, USA

During my final rotation of medical school, I cared for an older woman with severe aortic stenosis and chronic kidney disease. She was an extremely pleasant woman – kind, thoughtful, grateful – much like her husband and son, who visited daily. But her physiology was tenuous. Too much fluid and her kidneys would lag behind and her lungs fill with water. Too little hydration and her heart would not squeeze enough to replenish vital organs. Each day was a tightrope juggling act of kidney function, cardiac output, hemodynamics, and electrolyte derangements.

After a week of little progress, I organized a family meeting. We spoke for over an hour, describing the fragile physiology that constrained us. The patient drifted in and out of the conversation, her senses dulled by rising uremia. We could try more diuresis, I said, and potentially ease her breathing. We might also cause damage, drying up what little fluid she had remaining. I touched on the possibility that there was no true fix to her problems. But if there was no fix – ?

I told them about hospice and comfort care. We were not there yet. But we could be there shortly. After a long conversation, we decided to try another round of diuresis and watch her kidney function and mental status. It would take a few days to know whether this had worked.

To a certain degree, it did. Her kidney function improved, her cardiac output appeared intact, and she was more alert. This was not a total victory, but her husband and son were grateful. I had only 5 or 6 patients on my list, so I spent hours with them as my rotation wound down. They showed me photos from her youth, told me I was her favorite doctor. The son smiled when I walked in the room. I returned each night after sign-out to answer their questions or call the husband if I hadn’t seen them in the afternoon.

That was the case on my last day. After a warm goodbye with my attending, I went to the patient’s room and found her asleep, by herself, the uremia making her hard to wake, her seesaw physiology swinging back from a brief reprieve. I didn’t want to bother her, so I left a short note to be read when she woke up. This may never have happened. I left the hospital and walked in the warm early-summer air, grabbed my bike, and cycled to my dormitory.

Around this time, I had been reading quite a lot of Ivan Illich, the 20th-century social critic, Catholic priest, university professor, and public intellectual, perhaps most famous (certainly among clinicians) for his 1976 publication, *Medical Nemesis*, later updated to *Limits to Medicine* (O’Mahony 2016). Illich was not without his flaws, but he drew me in with his provocative and frankly polemical critiques of the “medical establishment,” which he saw as one of several modern institutions whose activities constituted a “paradoxical counterproductivity” – an active undermining of their original aims (Illich 1976). Among these institutions he also included mass obligatory schooling, modern transport systems, and contemporary modes of energy production and distribution (Illich 1970, 1973, 1974). At the center of Illich’s critique was the idea that human beings had a natural capacity to learn, move, build, heal, and face death, and to do this for themselves and within communities of mutual support and personal interdependence.

What Illich criticized was the increasing tendency to “expropriate” these activities as the products of impersonal institutions. In each of his major works – which exerted great influence at the time, and were reviewed by the likes of the *New York Times* and *New York Review of Books* – he questioned not the existence but rather the unquestioned centrality and expansion of hospitals, highways, and school certificates. These things could had their place in society, but their ideal use, in Illich’s view, was not toward constant growth but rather *conviviality*. He defined this as “individual freedom realized in personal interdependence,” a reciprocal growth in autonomy and community, in which tradition and shared knowledge contributed to each person’s ability to care for themselves and for their neighbor.

One of his favorite parables was that of the Good Samaritan, from the Christian New Testament (Cayley 1992, 2021). In the parable, a man is beaten and left for dead on the side of the road, unaided by passing strangers from his tribe. A man from a rival tribe, the Samaritans, stops and binds his wounds and pays for his recovery in a nearby inn. The Samaritan, as Illich pointed out, is not directed by any ethical imperative – individuals from the two people groups were not expected to assist one another in this way – but rather by compassion, by being “moved in his guts” (Cayley 1992, 2021).

Jesus delivers the parable in the New Testament in response to a question about the imperative to love one’s neighbor as oneself. Who is this neighbor? To whom do we owe this love? The answer is more complicated than the usual interpretation. By assisting a man from an opposing tribe, the logic goes, the Samaritan shows that *everyone* is the neighbor. But Illich argued that the Samaritan is not obeying any ethical code when he decides to help the man left for dead on the side of the road. He is moved in his guts, gratuitously touched on a physical and emotional level. The man on the road is not an abstraction or an ethical case study, but a human being who has the potential to *become* the neighbor in the process of mutual care and recognition. The Samaritan has the ability to *choose* whom he will recognize as a neighbor, in a way that cannot be prescribed by codes or institutions.

It is this spirit of autonomy, interpersonal dependence, and gratuitous generosity that Illich wanted for modern medicine. He claimed that the growth of medicine into a massive industry had interfered with its convivial potential, and that this turned care into a commodity rather than a human activity, a noun rather than a verb (Illich 1973). He was a “proscriptive” rather than a “prescriptive” thinker, as David Cayley argues, and did not devise a detailed solution to this problem. As Lewis Thomas wrote in his review of *Medical Nemesis*, “it is possible to read the whole book through, nodding much of the time in general agreement,” but another issue arises “when it is finished … and you try to figure out what Illich wants to have done about it” (Thomas 1976).

And yet, despite these limitations, I returned to Illich after saying goodbye to my patient, and I continue to return to him during my internship. I have seen a good deal of pain and death in the last several years, much of which has happened in the hospital and thus within the purview of a massive and highly specialized caregiving institution. Almost all of it has gone far beyond the caregiving capacity of loved ones who must continue to work, raise families, and sleep. Even with the rise of palliative care interest and training, and the consequent attention to goals of care and the avoidance of unnecessary intervention, we therefore retain the possibility of non-convivial care, of delivering a product instead of fostering human activity. We attend closely to patient-centered care, including patients and their families in our decision-making, as I did during the family meeting for my patient. But I wonder what it would have meant to make that care convivial, to help my patient and their family care for one another, not only to provide care to them. Maybe I achieved this. Maybe partially.

The first time I read Ivan Illich, I mistook his name for a misspelling of Ivan *Ilyich*, the protagonist of a Tolstoy novella (Tolstoy et al. 2010). Years ago, when I was a hospice volunteer before medical school, I published an article in this journal about that novella (Petriceks 2019). I focused on Gerasim, a young peasant, as perhaps the sole caregiver whom the fictional Ivan Ilyich genuinely appreciates. Gerasim evokes this appreciation through “honesty, authenticity, and humility,” I wrote, and this is true, but as a doctor, I now see Gerasim in a different light. He embodies all those traits, but he is also slow, gracefully limited, equipped with his own physical ability to carry and comfort Ivan Ilyich. There is no abstract professional identity or ethical imperative guiding him. He responds gratuitously to the suffering man who enters his life. He is caring, compassionate.

In a word, convivial.
